# The complete mitochondrial genome of *Metidiocerus impressifrons* (Kirschbaum, 1868) (Hemiptera: Cicadellidae: Idiocerinae)

**DOI:** 10.1080/23802359.2021.2008835

**Published:** 2022-02-15

**Authors:** Mingrui Ma, Zhengnan Li, Xianguang Guo, Bin Zhang

**Affiliations:** a College of Life Sciences & Technology, Inner Mongolia Normal University, Hohhot, China; b College of Horticulture and Plant Protection, Inner Mongolia Agricultural University, Hohhot, China; c Chengdu Institute of Biology, Chinese Academy of Sciences, Chengdu, China

**Keywords:** Leafhopper, *Metidiocerus impressifrons*, mitochondrial genome, phylogenic relationship

## Abstract

We determined the complete mitochondrial genome (mitogenome) of the leafhopper *Metidiocerus impressifrons* by next-generation sequencing. The mitogenome sequence was 16,426 bp in length and consists of 13 protein-coding genes, 22 transfer RNA (tRNA) genes, 2 ribosomal RNA (rRNA) genes, and a control region. Moreover, the nucleotide composition biases toward A and T, which together made up 78.2% of the entirety. The complete mitochondrial genomes of *Metidiocerus impressifrons* and other 27 species were used for phylogenetic analysis using the Bayesian method. The above results would facilitate our understanding of the evolution of Idiocerinae mitochondrial genome.

The leafhopper subfamily Idiocerinae is the largest arboreal leafhopper in the world, and many species are important economic pests, there are nearly 800 species in 106 Genera in the world (Zhang and Webb 2019). The taxonomic position of *Metidiocerus impressifrons* (Kirschbaum 1868) belongs to the Class: Insecta, Order: Hemiptera, Family: Cicadellidae, Subfamily: Idiocerinae, Genus: *Metidiocerus*. The genus *Metidiocerus* was originally described by Ossiannilsson containing six species and distributed only in the Palearctic region. Kwon (1985) considered *Metidiocerus* as a subgenus of *Idiocerus* and described one new species, *Idiocerus (Metidiocerus) nigrolineatus*. Subsequently, Anufriev elevated it to a separate genus. Studies of its phylogeny have important implications for biodiversity, the origin and evolution of arthropods, and the study of zoogeography.

In this study, the complete mitochondrial genome of *M. impressifrons* was first sequenced and described, with GenBank accession number MW963341. The specimens of *M. impressifrons* were collected on 27 July 2020 in Tuquan County, Inner Mongolia (45.581389°N, 121.44162985°E). The samples and voucher specimens (IMNU2020072708) were stored in Inner Mongolia Normal University Museum, China. The entire body without abdomen was shipped to Tsingke Biotechnology Co., Ltd. (Beijing, China) for genomic extraction. The mitogenome sequence of *M. impressifrons* was generated using Illumina HiSeq Sequencing System. The resultant reads were assembled using the SPAdes v3.13.0 (Bankevich et al. 2012). The complete mitochondrial genome was annotated with MITOS2 (Bernt et al. 2013). The sequences were analyzed by MEGA7 (Tamura et al. 2013). tRNA genes and PCGs were annotated by aligned with homologous genes from Cicadellidae species.

The mitochondrial genome of *M. impressifrons* possesses 16,426 bp and the base nucleotide composition is 41.3% A, 36. 9% T, 11.6% C, and 10.2% G, with an A＋T (78.2%) rich feature. It is within the range reported for hemipteran mitogenomes(68.86–86.33%; Zhang et al. 2014). The 22 tRNA genes begin from 63 bp (tRNA^A, S2^) to 72 bp (tRNA^K^), while 16S rRNA possesses 1188 bp and 12S rRNA possesses 742 bp in length. ATskew is 0.06 and GCskew is −0.06, showing a significant A deviation and a significant C deviation. The length of the protein coding gene is 10,917 bp, the base composition is A: 32.3%, T: 43.5%, C: 11.7%, G: 12.4%, A + T content is 75.9%, which is much higher than G + C content and has obvious A + T preference. The starting codon of the 13 protein coding genes was mostly ATN, only ATP8 and ND5 were TTG start codon; most of the 13 protein coding genes were TAA, and the special COI was incomplete TA termination and COII, ND5, ND4, ND1 were incomplete T termination.

In order to assess its phylogenic relationship and evolution of different species, all 13 PCGs sequences were extracted from the mitochondrial DNA sequences of 27 related taxa of Cicadellidae and an outgroup. The concatenated PCGs using Bayesian inference (BI) method in MrBayes v3.2.1 (Ronquist and Huelsenbeck 2003) under the GTR + G model. The phylogenetic tree (Figure 1) showed that *M. impressifrons* was clustered into the subfamily Idiocerinae. This is consistent with previous research (Di et al. 2020). The complete mitogenome of *M. impressifrons* will promote future phylogenetic study of Cicadellidae.

**Figure 1.**
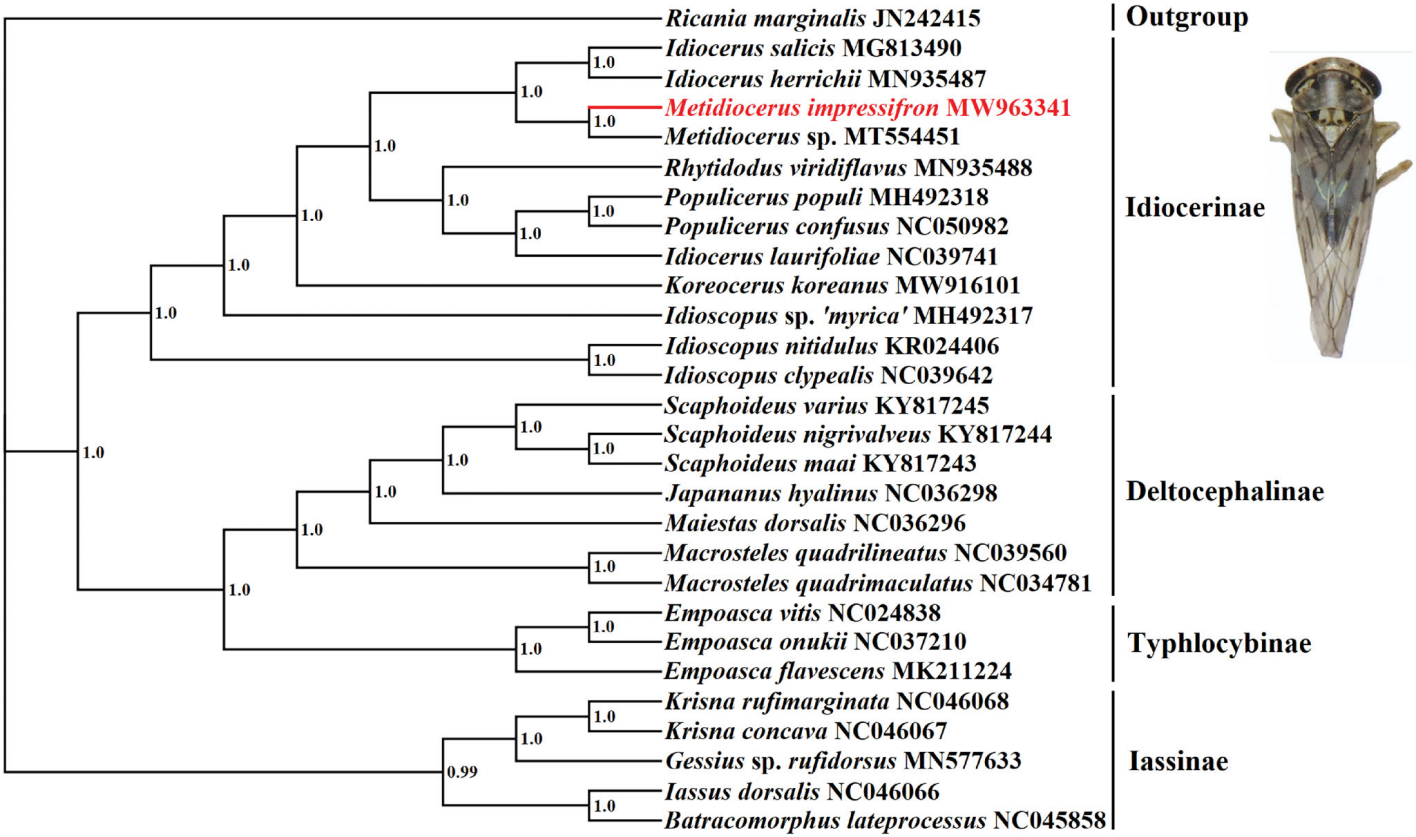
Phylogenetic tree of the relationships among 28 species of based on the nucleotide dataset of 13 PCGs. Numbers above the nodes indicate the posterior probabilities of Bayesian inference using MrBayes v3.2.1 under the GTR + G model. Branch lengths represent means of the posterior distribution. The GenBank numbers of all species are shown in the figure.

## Data Availability

The genome sequence data that support the findings of this study are openly available in GenBank of NCBI at https://www.ncbi.nlm.nih.gov/ under the Accession no. MW963341. The associated BioProject, SRA, and Bio-Sample numbers are PRJNA741095, SRR14925696, and SAMN19844819, respectively.
